# En bloc resection of total thyroid and bilateral central compartment lymph nodes *via* a gasless transoral approach in papillary thyroid carcinoma

**DOI:** 10.3389/fendo.2023.1130791

**Published:** 2023-02-27

**Authors:** Xuren Sheng, Jianjun Liu, Jing Fang, Xucai Zheng, Shengying Wang

**Affiliations:** ^1^ Department of Head and Neck Surgery, West District of The First Affiliated Hospital of University of Science and Technology of China, Division of Life Sciences and Medicine, University of Science and Technology of China, Hefei, China; ^2^ Department of Head and Neck Surgery, Anhui Provincial Cancer Hospital, Hefei, China

**Keywords:** en bloc resection, papillary thyroid carcinoma, gasless, transoral, total thyroid

## Abstract

**Introduction:**

The current study presents a preliminary exploration of en bloc resection via a gasless transoral approach in papillary thyroid carcinoma.

**Objective:**

This study aimed to summarize and explore the efficacy and safety of en bloc resection of total thyroid and bilateral central compartment lymph nodes *via* a gasless transoral approach in patients with papillary thyroid carcinoma.

**Methods:**

This study was conducted between January 2021 and December 2021. It involved 30 patients with bilateral papillary thyroid carcinoma who had undergone en bloc resection of the total thyroid and bilateral central compartment lymph nodes *via* a gasless transoral approach using a three-trocar and four-instrument technique at The First Affiliated Hospital of the University of Science and Technology of China. The key steps and difficulties of the operation were summarized, and the clinicopathological characteristics and surgical complications of patients were analyzed.

**Results:**

All operations were successful without conversion to open surgery. The pathological diagnosis was bilateral papillary thyroid carcinoma. The mean maximum tumor diameter was 0.85 ± 0.51 cm (range 0.3–2.5 cm). There was no case of gross capsular invasion. The mean number of harvested central compartment lymph nodes was 11.36 ± 5.36. Central compartment lymph node metastases were found in 16 patients (53.3%) with a mean of 1.53 ± 2.39. On the other hand, lymphocytic thyroiditis was observed in 12 cases (40%), and microscopic capsular invasion was observed in five cases (16.6%). All patients had normal parathyroid hormone levels after the operation. However, one patient developed hoarseness after the operation due to injury of the recurrent laryngeal nerve branch, but there was no numbness of the mandible and lower lip or infection of the oral incision.

**Conclusion:**

The study revealed that the three-trocar and four-instrument technique can be used in the en bloc resection of total thyroid and bilateral central compartment lymph nodes *via* a gasless transoral approach without disconnecting the thyroid isthmus. As a result, the operation is considered effective and safe. Therefore, this technique may be a better surgical method for patients with bilateral thyroid cancer and cosmetic needs.

## Introduction

An epidemiological survey of 36 cancers in 185 countries found that thyroid cancer is one of the most prevalent malignant diseases worldwide, with 586000 new cases per year (1). The survey also reported that surgery is one of the most important treatment methods for thyroid cancer. Patients with papillary thyroid cancer have a long survival period after surgery (2). Many patients have demands regarding the cosmetic effect of the incision. It has been reported that transoral thyroidectomy is safe, with favorable cosmetic outcomes when compared to minimally invasive video-assisted thyroidectomy (3). As a result, surgical experts worldwide use transoral endoscopic thyroidectomy vestibular approach (TOETVA) because it yields excellent oncological results and good cosmetic effects. The use of TOETVA has been reported in Thailand, Korea, China, India, the United States, Mexico, Japan, the Philippines, Indonesia, Ecuador, Italy, etc. (4). Reviewing published studies on endoscopic thyroid surgery revealed that these studies are mostly limited to benign thyroid diseases, such as benign thyroid tumors or Graves’ disease (5–9). To date, the study with the largest sample size was reported by Anuwong A, who utilized 422 participants and only 26 patients with thyroid cancer (10). On the other hand, Kim SY et al. evaluated 132 participants with thyroid cancer treated with TOETVA. However, only eight participants received total thyroidectomy and bilateral central lymph node resection. Moreover, the researchers did not mention en bloc resection (11). In endoscopic thyroid surgery, surgeons usually perform central compartment lymph node dissection after tumor resection due to the relatively narrow operation space and unskilled operation. There are several reports of tumor implantation metastasis after endoscopic thyroidectomy (12–17). En bloc resection emphasizes the removal of the primary tumor, adjacent normal tissue, and lymphoid adipose tissue as a whole, with the aim of avoiding tumor fragmentation and implantation metastasis. Moreover, traditional CO_2_ inflatable endoscopic surgery has several complications, including CO_2_ embolism, which make the surgery ineffective and life-threatening (18–21). To avoid this situation, domestic and foreign scholars have explored gasless endoscopic thyroid surgery (22–27). No studies have been conducted on en bloc resection of the total thyroid and bilateral central lymph nodes *via* a gasless transoral approach in papillary thyroid carcinoma. The current study presents a preliminary exploration of en bloc resection *via* a gasless transoral approach in papillary thyroid carcinoma.

## Materials and methods

### Clinical data

This retrospective study was performed in the Department of Head and Neck Surgery at The First Affiliated Hospital of the University of Science and Technology of China. In this study, 30 patients received en bloc resection of the total thyroid and bilateral central lymph nodes *via* a gasless transoral approach between January and December 2021. All patients were diagnosed with bilateral papillary thyroid carcinoma using aspiration cytology before the operation. Laryngoscopy showed no paralysis of the vocal cords. All patients had normal functioning of the thyroid. All operations were performed by a single surgeon. Operative time was defined as the time from the initial skin incision to the point of final closure. This study was approved by the hospital ethics committee. The follow-up time was 6 months in this study.

### Instruments required for surgery

**Figure 1:**
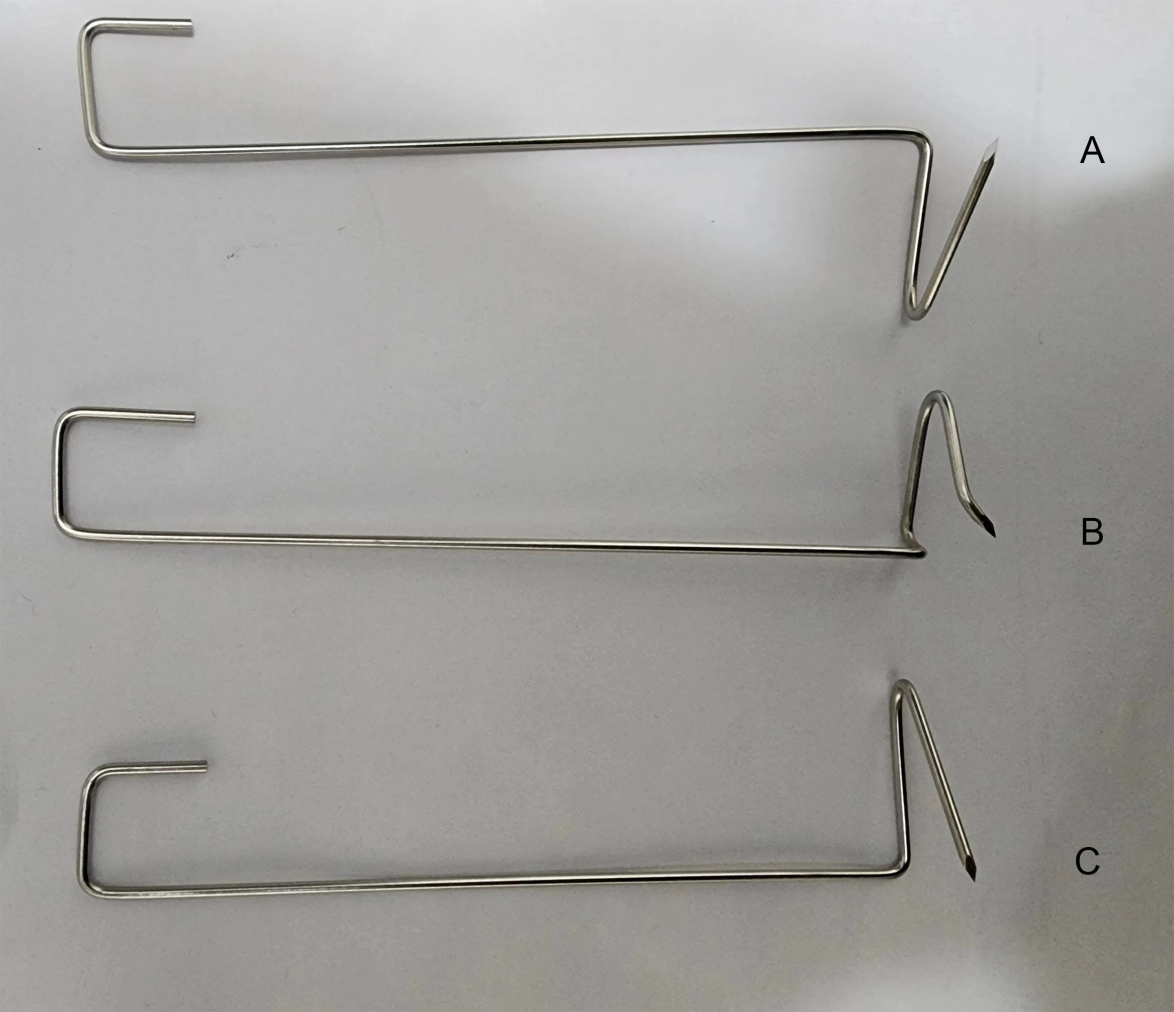
The self-designed right thyroid retractor. **(A)** The self-designed skin suspension hook. **(B)** The self-designed left thyroid retractor. **(C)**.

The three self-designed trocars (two operational trocars, one observational trocar) and thyroid retractor used were similar to those reported in our previous study (26). However, unlike our previous study, this study involved bending the Kirschner wire into a hook at a specific angle, named the skin suspension hook (**Figure 1**).

### Surgical procedures

**Figure 2:**
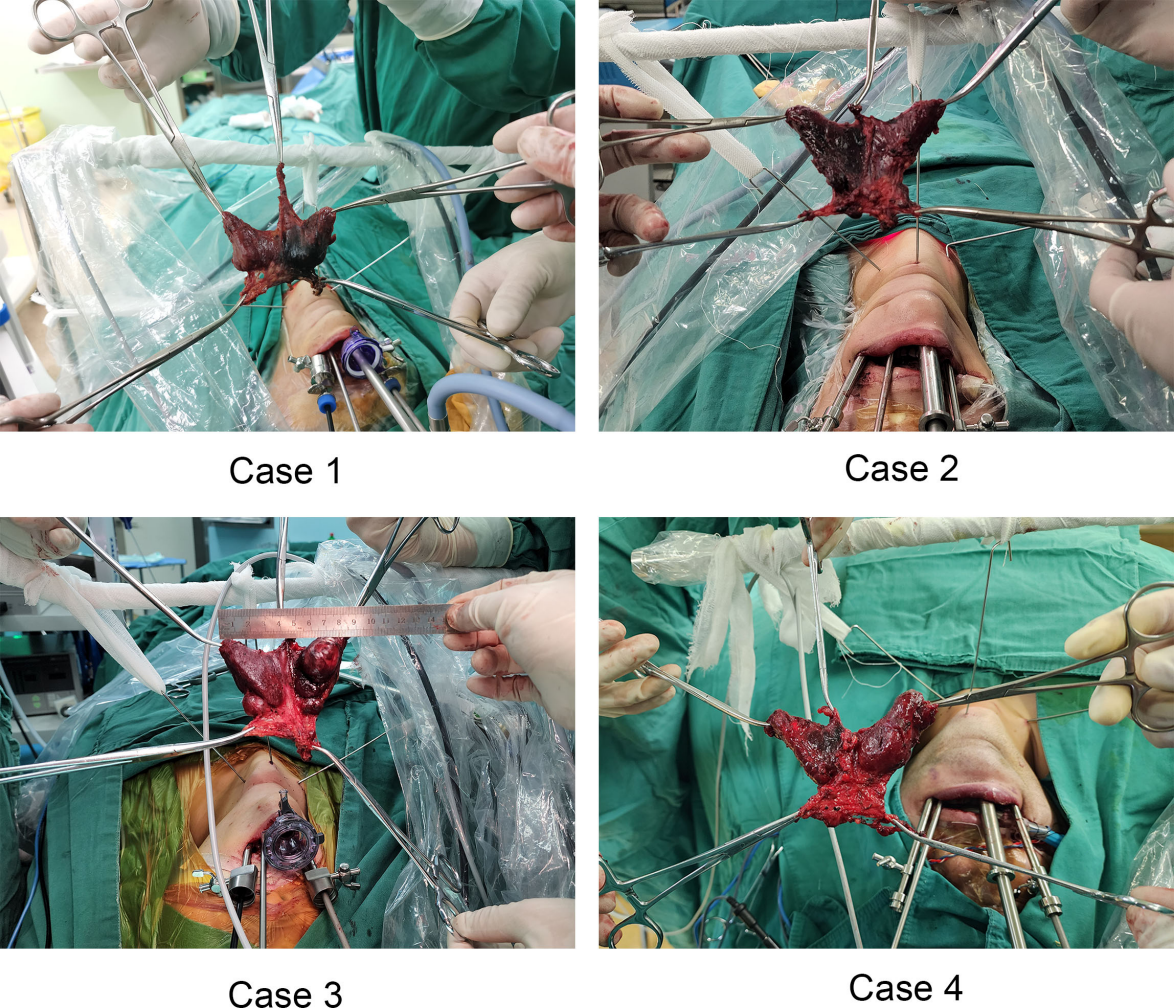
The en bloc resected total thyroid and bilateral central compartment lymph nodes.

**Figure 3:**
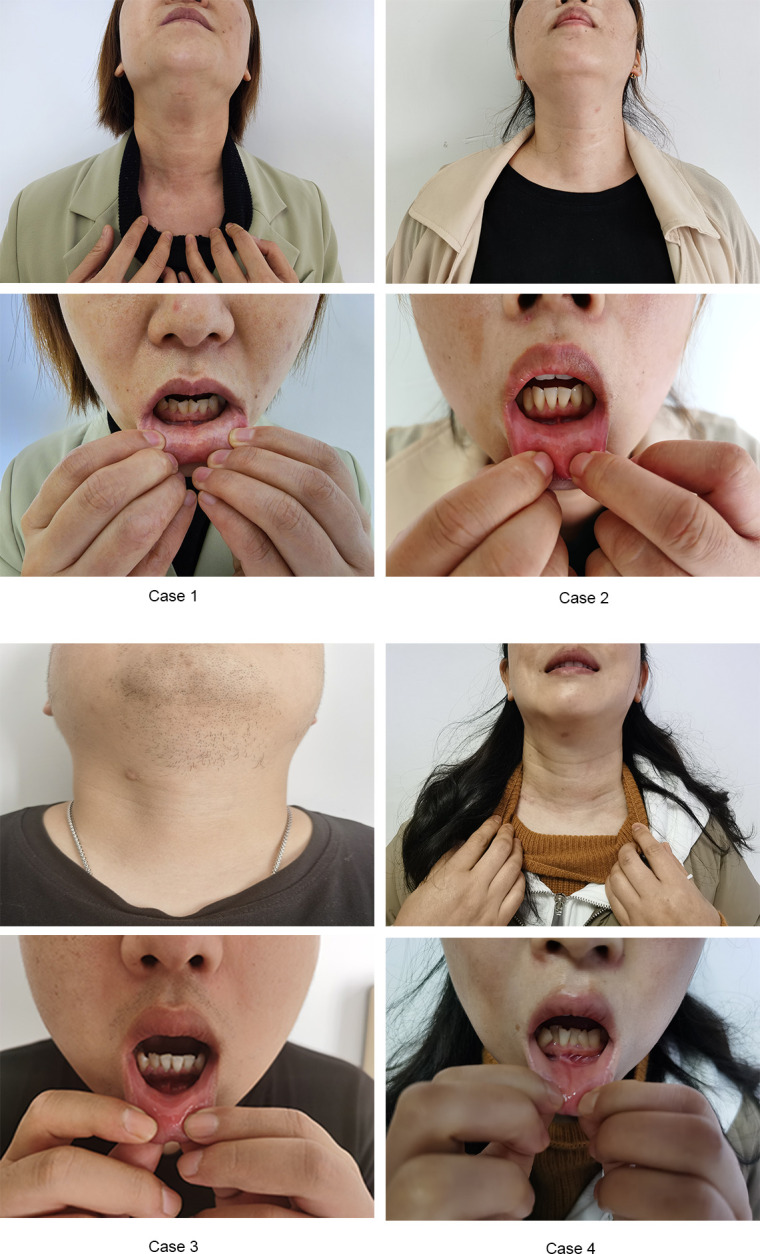
Patients’ front view of the oral vestibule incisions and neck after surgery.

The posture and anesthesia used in this study were similar to those utilized in our previous study (26). The surgery was performed in two steps. The first step involved establishing the operation space. However, we improved the positions of the observational and operational ports used in the previous study. The observational port was located in front of the lower lip frenulum and 15 mm from the gingival root. The two operational ports were located at the buccal mucosa of the first premolars on both sides (near the lip). Since the three trocars were directly converged near the laryngeal nodes and the correct layer was found and slightly separated, the self-designed skin suspension hook was placed through the percutaneous puncture and fixed in the head frame of the operating room with bandages. Thus far, the operation space had a stable suspension support, the smoke was removed efficiently, the space was stable and did not collapse, and the operation could be carried out smoothly. Step two involved en bloc resection of the total thyroid and bilateral central compartment lymph nodes. The first step in en bloc resection was to divide the midline of the strap muscles to expose the thyroid gland. The anterior Delphian lymph nodes were swept, and the vertebral thyroid lobe was removed. It has been recommended that right-handed surgeons should first treat the right thyroid lobe. A self-designed thyroid retractor was inserted into the medial edge of the sternocleidomastoid muscle under direct endoscopic vision and fixed in the head frame, which was mentioned in our previous study (26). Next, the suspensory ligament of the thyroid was cut to allow entry into the cricothyroid space. Next, part of the sternal thyroid muscle was cut to expose the upper pole of the thyroid. To avoid injury to the superior laryngeal nerve, the upper pole blood vessels of the thyroid were cut close to the gland. The separation continued along the dorsal side of the upper pole of the thyroid. In addition, the superior parathyroid gland was dissected and retained in situ. With the aid of intraoperative neural monitoring (IONM), this study identified the recurrent laryngeal nerve (RLN) at the RLN laryngeal entry point. The separation proceeded downward to observe whether there were anterior and posterior branches. The Berry ligament of the thyroid gland was disconnected, exposing the RLN during the process. Then, the inferior parathyroid gland was identified and protected. The right central lymph nodes were dissected and the thymus was preserved. At this point, the right thyroid gland lobe and the surrounding lymphatic adipose tissue were en bloc resected and dissociated to the opposite side to expose the trachea. The left thyroid gland lobe and central lymph nodes were removed in the same way. After en bloc resection of the total thyroid and bilateral central lymph nodes, the resected specimen was placed into the bag and moved outside the body through the observation incision. The surgical wound was washed with 1000 ml distilled water, and only one drainage tube with a diameter of 3 mm was placed in the thyroid bed and led out from the submental area. Postoperative management followed according to the routine TOETVA **Figures 2**, **3**.

## Results

All operations were successful without conversion to open surgery. All the patients in this study had confirmed bilateral papillary thyroid carcinoma in the pathologic report. The mean age of the patients in this study was 38.06 ± 10.76 years, and the male-to-female ratio was 1:29 (1:29 patients). The mean maximum tumor diameter was 0.85 ± 0.51 cm (range 0.3–2.5 cm). There was no case of gross capsular invasion. The mean number of harvested central compartment lymph nodes was 11.36 ± 5.36. Central compartment lymph node metastases had a mean of 1.53 ± 2.39 and were found in 16 patients (53.3%). On the other hand, lymphocytic thyroiditis was observed in 12 patients (40%), and microscopic capsular invasion was observed in five patients (16.6%). The mean operation time was 192.86 ± 43.39 minutes, the mean bleeding volume was 34.66 ± 12.97 mL, the mean drainage volume of the entire period was 257.66 ± 77.90 mL, and the mean drainage tube removal time was 5.9 ± 1.19 days. All patients had normal parathyroid hormone levels after the operation. There was no numbness of the mandible and lower lip or infection of the oral incision. However, one patient developed hoarseness after the operation due to recurrent laryngeal nerve branch injury (**Table 1**).

**Table 1:** Clinicopathologic characteristics of patients (n = 30).

Variables	Mean ± SD/Number	Range/Percent
Age (years)	38.06 ± 10.76	20-64
Sex		
Male	1	3.33%
Female	29	96.66%
Maximum tumor size (cm)	0.85 ± 0.51	0.3-2.5
Harvested central compartment nodes	11.36 ± 5.36	3-20
Central compartment lymph node metastases	1.53 ± 2.39	0-9
Thyroiditis	12	40%
Microscopic extrathyroidal extension	5	16.6%
Operation time (min)	192.86 ± 43.39	110-298
Bleeding volume (ml)	34.66 ± 12.97	20-80
Drainage volume of the entire period (ml)	257.66 ± 77.90	115-440
Drain removal (days)	5.9 ± 1.19	3-9
Lesions of RLN	1	3.33%
Hypoparathyroidism	0	0%
Surgical site infection	0	0%
Numbness of the lower lip	0	0%

## Discussion

Differentiated thyroid cancer is the most common thyroid cancer, accounting for more than 95% of cases, of which papillary thyroid cancer is the most common subtype (28). It has been reported that up to 40% of patients with differentiated thyroid cancer have central compartment lymph node metastasis, and up to 80% have undetectable micro-metastases in central compartment lymph nodes (29). Previously, the occult metastatic rate of central compartment lymph nodes detected in prophylactic central neck dissection (pCND) was 24% to 82% (30). It has also been reported that lymph node metastasis is associated with a higher risk of recurrence in papillary thyroid cancer (31). Previous studies have shown that pCND has a positive effect on patient survival, reducing the probability of regional recurrence (32, 33). However, most studies show that pCND prevents the occurrence of regional nodal recurrences, but does not provide any clear benefit in long-term patient survival (34–36). The indication of pCND is still a controversial issue. It has also been reported that the performance of pCND is indicated in patients with multicenter thyroid carcinomas (37). It needs to be explained that this study included 19 cases of bilateral papillary thyroid carcinoma measuring less than 1 cm in the maximum diameter. Among these, 3 cases were butting up against the trachea, 5 cases located at the back of the thyroid, and 15 cases were indicated to have central lymph node metastasis by imaging examination. These high-risk factors determined the surgical decision-making instead of active surveillance or percutaneous ablation. In this study, all cases were pathologically confirmed as bilateral papillary thyroid carcinoma with a total number of lesions ≥ 2. All patients underwent en bloc resection of the total thyroid and bilateral central compartment lymph nodes *via* a gasless transoral approach, which is more in line with the non-touch isolation technique of tumor surgery. Theoretically, this reduces the risk of recurrence and reoperation in the central compartment.

En bloc resection of the total thyroid and bilateral central compartment lymph nodes is difficult, especially under an endoscope. The difficulties are mainly reflected in the following three points: establishment and maintenance of the surgical space, auxiliary exposure during the operation and obstruction of the thyroid gland during central compartment neck dissection, and protection of the parathyroid gland and recurrent laryngeal nerve. With regard to the application of en bloc resection of the total thyroid and bilateral central compartment lymph nodes *via* a gasless transoral approach, our center has adopted the following operation protocols. First, en bloc resection of the total thyroid and bilateral central compartment lymph nodes requires a sufficiently continuous stable operating space to meet the turnover and movement of the thyroid gland in the operation space. Surgeons are required to expose the anterior edge of the sternocleidomastoid muscle on both sides during the establishment of the surgical space. It is recommended that the lower boundary should exceed the superior fovea of the sternum. The self-designed skin suspension hook can maintain a stable operating space during the operation. Unlike the suture suspension reported by Kim SI (38), Peng X (39), Moreno Llorente P (40) et al., the space maintained by the self-designed skin suspension hook is stable and three-dimensional. As a result, it can be rotated according to the needs of surgery, and the suspension space can be adjusted without adding pinholes. Second, The First Affiliated Hospital of the University of Science and Technology of China innovatively proposed the three-trocar and four-instrument technique. In fact, this technique was only used in our previous research (26). After continuous improvement, this concept was first proposed in this research. The three-trocar and four-instrument technique means that an endoscopic aspirator is added on the left side of the observational trocar, and one instrument is added based on the original three instruments. The thyroid gland may shield the central compartment lymph nodes during en bloc resection of the total thyroid and bilateral central compartment lymph nodes, resulting in poor exposure of the central compartment lymph nodes. Poor exposure of the central compartment lymph nodes makes it difficult to dissect the central compartment neck. To solve this problem, an assistant should hold the endoscopic aspirator, pick up the thyroid gland or push the trachea to assist in exposing the central compartment lymph nodes. This exposes the central compartment lymph nodes allowing the surgeon to remove the central compartment lymph nodes. The thymus tissue may block the front of the field of vision when dissecting the lower boundary of the central compartment. In such a case, the assistant can use the endoscopic aspirator to pick up the thymus. The chief surgeon can then hold the separating forceps with his left hand and use the ultrasonic scalpel with his right hand to penetrate the rear of the thymus and remove the lymph nodes. While assisting exposure, the endoscopic aspirator near the energy instrument can quickly discharge the smoke generated during surgery, making the surgical field of vision clear. Therefore, there is no need to wipe the lens repeatedly during surgery. Unlike the four-trocars method proposed by Ngo DQ et al. (41), the endoscopic aspirator used in this study does not interfere with other surgical instruments. On the premise of not increasing the incision length, the endoscopic aspirator can assist in exposure, remove smoke, attract blood and exudate during surgery, which is the fourth instrument with both smoke extraction and auxiliary exposure. Adding an endoscopic aspirator as an independent instrument made the surgical process smooth. The mean operation time in this study was 188.92 ± 46.04 minutes. This operation time was shorter than that in a previous study at The First Affiliated Hospital of the University of Science and Technology of China (26). Moreover, the extent of surgery in this study was larger and the procedure more difficult than that in our previous study. Finally, total thyroidectomy requires better protection of the parathyroid gland and RLN. In this study, one patient developed temporary postoperative hoarseness caused by an RLN branch injury. Since the RLN is anatomized retrogradely at the RLN laryngeal entry point, IONM can be used during the operation. After exposing the RLN at the RLN laryngeal entry point, it is still necessary to continue downward separation. The Berry ligament should only be disconnected when the RLN is exposed in the process and when it is confirmed that there are no anterior and posterior branches. This study revealed that the patients’ parathyroid hormone levels were within the normal range one month after the operation. Nanocarbon-negative imaging can be used during the operation to protect the parathyroid gland. In addition, the parathyroid gland can be protected by retaining it *in situ* and paying special attention to protecting its blood supply.

There are three innovations of this study. First, en bloc resection of the total thyroid and bilateral central compartment lymph nodes was performed successfully without disconnecting the isthmus. This technique is more in line with the non-touch isolation technique of tumor surgery. Theoretically, this technique prevents implantation metastasis caused by the spread of cancer cells and has a good oncological effect. Second, based on not increasing the incision length of the observational trocar, an endoscopic aspirator was added on the left side of the observation trocar as an independent instrument, which is called the three-trocar and four-instrument technique. The increased endoscopic aspirator can attract accumulated blood, liquid and smoke and assist in exposing the central compartment lymph nodes. It is an indispensable third hand for the chief surgeon and can effectively assist in completing complicated endoscopic surgery, such as gasless endoscopic-assisted lateral neck lymph node dissection. Third, a special skin suspension hook was designed to maintain the surgical space. The self-designed skin suspension hook has a wide hanging force-bearing surface, and the maintenance of the surgical space is larger. We have a little skill about the placement of the skin suspension hook and the thyroid retractor. When the endoscopic light source was closed to the skin, the subcutaneous blood vessels can be clearly observed. Then we punctured the skin with the tip of the hook. Care was taken to avoid the subcutaneous blood vessels. Finally, the hook was placed in the specified position under direct endoscopic vision. So far, no complications such as bleeding or hematomas have been found. The eye of the needle has only one pinhole with a diameter of 2 mm and no indentation. Unlike previously designed hooks, this suspension hook has better cosmetic effects. In this study, the wounds caused by the hook healed with almost no scarring. Like the thyroid retractor, the hook is made with Kirschner wire, and the manufacturing method is simple and easy for clinical popularization.

Although no complications such as infection, postoperative bleeding, hypoparathyroidism, numbness of the mandible and lower lip were found in our study, it is worth mentioning that the period of permanence of the drainage (3-9 days) was excessive. This may be due to the fact that we have performed en bloc resection of the total thyroid and bilateral central compartment lymph nodes, the extent of surgery in this study was larger and the central lymph node dissection was thorough, there were no residual lymphatic and adipose tissues. These factors led to more postoperative drainage. In addition, in order to prevent postoperative effusion in the operation area, our center is conservative in the removal of drainage tubes. In this study, one patient ‘s drainage tube was placed for nine days, which was a special case. The patient was very obese, with a weight of 160 kg and a BMI of 49.9. The patient had more postoperative drainage. Finally, the drainage tube was successfully removed without infection in the operation area. Since the diameter of the drainage tube was only 3 mm and the outlet was located in the submental area, the aesthetic effect was also good. In the future, we hope to improve this technique so that the drainage tube can be removed early or even not placed.

In conclusion, the three-trocar and four-instrument technique is feasible and safe for en bloc resection of the total thyroid and bilateral central compartment lymph nodes *via* a gasless transoral approach. It could be a better surgical method for patients with bilateral thyroid cancer and cosmetic needs. Therefore, this method may be worthy of clinical promotion. However, this study had a small sample size and a short follow-up time which cannot be used to make a conclusive decision. Therefore, future studies should involve large samples and a long follow-up duration.

## Data availability statement

The original contributions presented in the study are included in the article/**Supplementary Material**. Further inquiries can be directed to the corresponding author.

## Ethics statement

The studies involving human participants were reviewed and approved by Anhui Provincial Cancer Hospital ethics committee. The patients/participants provided their written informed consent to participate in this study. Written informed consent was obtained from the individual(s) for the publication of any potentially identifiable images or data included in this article.

## Author contributions

XS and JF conceived of the study. XS, JL, XZ and SW participated in its design and data analysis and statistics. XS and JL drafted the manuscript. All authors contributed to the article and approved the submitted version.

## Supplementary material

Supplementary Video Important organs view after en bloc resection of the specimen during gasless TOETVA.
